# Caveolin-1-Enhanced Motility and Focal Adhesion Turnover Require Tyrosine-14 but Not Accumulation to the Rear in Metastatic Cancer Cells

**DOI:** 10.1371/journal.pone.0033085

**Published:** 2012-04-10

**Authors:** Hery Urra, Vicente A. Torres, Rina J. Ortiz, Lorena Lobos, María I. Díaz, Natalia Díaz, Steffen Härtel, Lisette Leyton, Andrew F. G. Quest

**Affiliations:** 1 Laboratory of Cellular Communication, Center for Molecular Studies of the Cell, Faculty of Medicine, Universidad de Chile, Santiago, Chile; 2 Anatomy and Developmental Biology Program, Instituto de Ciencias Biomédicas, Faculty of Medicine, Universidad de Chile, Santiago, Chile; 3 Biomedical Neuroscience Institute, Instituto de Ciencias Biomedicas, Faculty of Medicine, Universidad de Chile, Santiago, Chile; 4 Department of Basic and Communitarian Sciences, Faculty of Dentistry, Universidad de Chile, Santiago, Chile; University of Toronto, Canada

## Abstract

Caveolin-1 is known to promote cell migration, and increased caveolin-1 expression is associated with tumor progression and metastasis. In fibroblasts, caveolin-1 polarization and phosphorylation of tyrosine-14 are essential to promote migration. However, the role of caveolin-1 in migration of metastatic cells remains poorly defined. Here, caveolin-1 participation in metastatic cell migration was evaluated by shRNA targeting of endogenous caveolin-1 in MDA-MB-231 human breast cancer cells and ectopic expression in B16-F10 mouse melanoma cells. Depletion of caveolin-1 in MDA-MB-231 cells reduced, while expression in B16-F10 cells promoted migration, polarization and focal adhesion turnover in a sequence of events that involved phosphorylation of tyrosine-14 and Rac-1 activation. In B16-F10 cells, expression of a non-phosphorylatable tyrosine-14 to phenylalanine mutant failed to recapitulate the effects observed with wild-type caveolin-1. Alternatively, treatment of MDA-MB-231 cells with the Src family kinase inhibitor PP2 reduced caveolin-1 phosphorylation on tyrosine-14 and cell migration. Surprisingly, unlike for fibroblasts, caveolin-1 polarization and re-localization to the trailing edge were not observed in migrating metastatic cells. Thus, expression and phosphorylation, but not polarization of caveolin-1 favor the highly mobile phenotype of metastatic cells.

## Introduction

Cell migration is essential in a large variety of biological processes, including embryonic development, tissue repair and regeneration, as well as events associated with diseases like arthritis, atherosclerosis and tumor cell metastasis [1]. Initially, cells respond to external cues (wounding, chemokines and growth factors) by reorientation of the microtubule organizing center (MTOC) and Golgi toward the leading edge (cell polarization step) [2]. Then, cells extend broad (*lamellipodia*) and spike-like protrusions (*filopodia*) in a process that is followed by focal adhesion (FA) formation, cell body contraction and finally retraction of the rear end by severing FAs [3]. Many of these events are organized by the Rho family of small GTPases, which includes RhoA, Rac1 and Cdc42. Signals that activate Rho GTPases lead to spatiotemporal regulation of FA assembly, dynamics and turnover [4], [5].

Many proteins including PI3K, PTEN [6], Rho-GTPases [4], [7] and caveolin-1 [8], [9], have been shown to re-distribute throughout the cell during polarized migration. Caveolin-1 is of special interest, since it is known to promote cell migration [10], invasion [11], [12], [13] and is proposed to be involved in tumor cell metastasis (reviewed in [14], [15]).

Caveolin-1 is an integral membrane protein involved in caveolae biogenesis, cholesterol homeostasis, intracellular trafficking and signal transduction, amongst other functions (reviewed in [15]). The precise role of caveolin-1 in tumorigenesis remains a matter of intense debate and whether the protein acts as a tumor suppressor or as a promoter of metastasis seems to be cell type and context-dependent [14], [15]. Thus, in cancer models including prostate, breast and colon, caveolin-1 expression is known to be up-regulated and protein levels correlate with tumor progression and metastasis [15]. Consistent with a role in metastasis, caveolin-1 was reported to enhance cell migration and invasiveness [11], [12], [16], [17]. However, the role of caveolin-1 during cell migration has been mostly characterized in non-metastatic cells. For instance, in mouse embryonic fibroblasts (MEFs), caveolin-1-promoted cell motility involved cell polarization and increased velocity and persistency of cell migration [18]. In addition, caveolin-1 decreases Rac1 and Cdc42, but increases RhoA activity by inhibiting the Src/p190RhoGAP signaling pathway [10], [18]. Intriguingly, caveolin-1 itself accumulates at the rear of migrating fibroblasts, neurons and endothelial cells in 2D models [19], [20], [21], although in transmigrating endothelial cells and neurons, caveolin-1 preferentially accumulates at the cell front [20], [22]. Caveolin-1-dependent cell migration and accumulation at the rear of MEFs require the aminoacid sequence 1-60, which encompasses a putative N-terminal caveolin-1 polarization domain [21], [23].

Caveolin-1 stabilizes the focal adhesion kinase (FAK) at FAs by decreasing the shuttling between FAs and cytosolic pools, as shown by fluorescence recovery after photobleaching analysis of the mobile fraction. Since FAK, and more specifically phospho-Y397-FAK, is known to enhance FA turnover, caveolin-1 was suggested to participate in the regulation of this process [24]. Indeed, subsequent studies showed that caveolin-1-dependent stabilization of FAK, cell migration and invasion involved the Src/Rho/ROCK axis as well as phosphorylation of caveolin-1 on tyrosine-14 [11]. Rather intriguingly, caveolin-1 was also shown to increase the stability of nascent FAs (or focal contacts) and RhoA activation [18].

Interestingly, Src kinase-mediated phosphorylation of caveolin-1 on tyrosine-14 appears to be essential for caveolin-1-driven cell migration. In metastatic breast cancer cells (MDA-MB-231), caveolin-1 is highly expressed and phosphorylated on tyrosine-14, in comparison to non-metastatic cancer cells [11]. Although initial studies suggested a putative role for caveolin-1 phosphorylation on tyrosine-14, controversial issues remain regarding the precise involvement of phospho-caveolin-1 in adhesion complexes with the extracellular matrix [25]. Most importantly, however, the role of caveolin-1 in migration of metastatic cells requires further investigation in order to understand how this protein may contribute to processes like metastasis.

In this study, we specifically investigated the role of caveolin-1 in metastatic cell migration by employing two different models: MDA-MB-231 human breast cancer cells and the mouse melanoma cell line B16-F10. In both cell lines, caveolin-1 promoted cell migration by increasing FA turnover, polarization, velocity, persistence and directionality. These functions of caveolin-1 are all blocked by interfering with phosphorylation on tyrosine-14, as shown by pharmacological inhibition and mutational analysis. Intriguingly, in both metastatic cell models, caveolin-1 localization to the cell rear was not required to promote migration.

## Results

### Caveolin-1 Accumulates at the Trailing Edge of Migrating MEF-3T3 and DI-TNC1 Cells, but not Metastatic MDA-MB-231 and B16-F10 Cells

**Figure 1:**
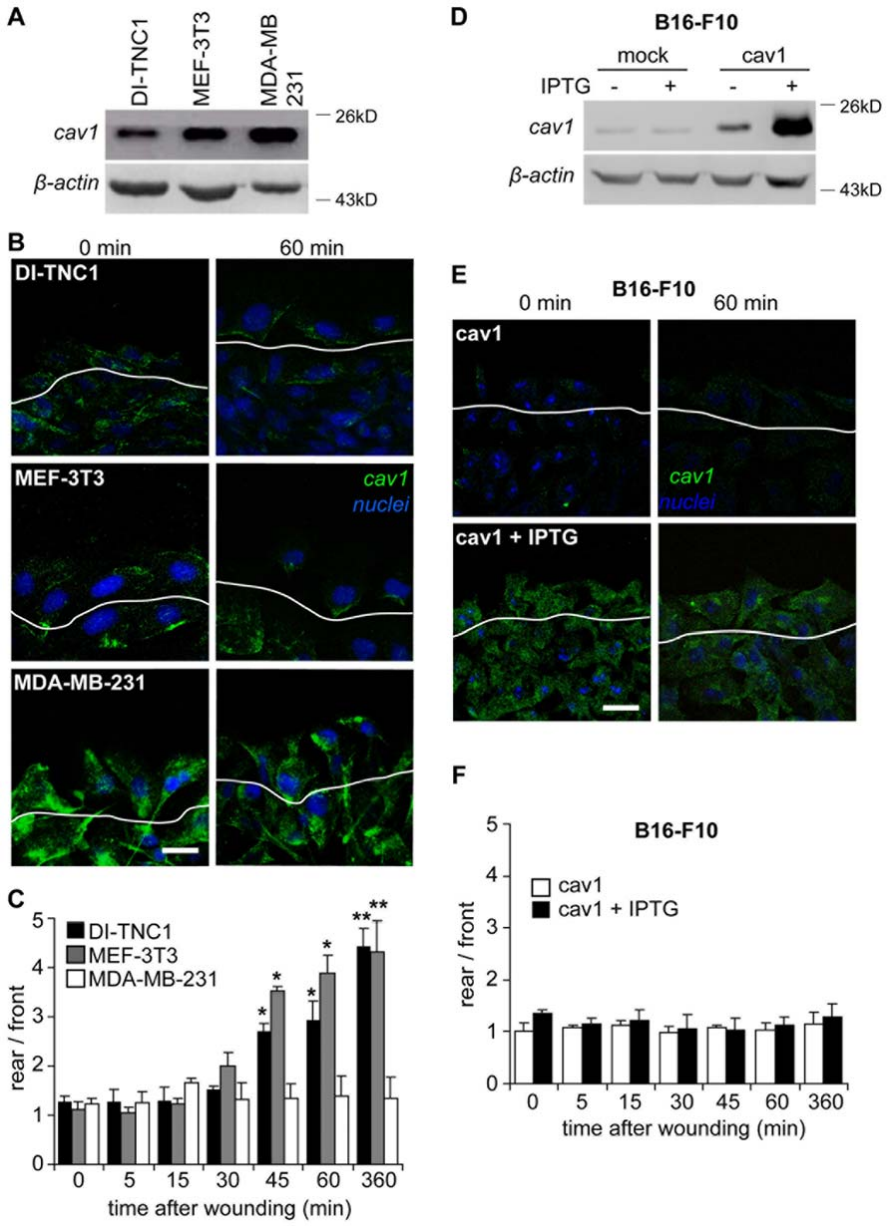
Caveolin-1 fails to accumulate at the trailing edge of migrating metastatic cells. (**A**) Total protein extracts from DI-TNC1, MEF-3T3 and MDA-MB-231 cells were separated by SDS-PAGE (35 µg total protein per lane) and analyzed by Western Blotting with antibodies against caveolin-1 and β-actin. (**B**) Confluent monolayers of DI-TNC1, MEF-3T3 and MDA-MB-231 cells were wounded with a pipette tip and fixed at 0 (left panel) and 60 minutes (right panel) after monolayer injury. Cells were stained with a rabbit polyclonal anti-caveolin-1 antibody followed by an anti-rabbit IgG antibody (green) and nuclei were visualized with DAPI (blue). The first layer of cells facing the wounded area is distinguished from the rest by a white line. Scale bar, 20 µm. (**C**) Caveolin-1 distribution was evaluated by measuring the fluorescence intensity in four randomly chosen regions of equal dimensions at the front and the rear of the cell with the *Image J* software, as detailed in the Materials and Methods. Data represent the ratio of fluorescence intensity “rear/front” (mean ± SEM; n =  3). Statistically significant differences compared with time 0 minutes for each cell type are indicated (**, P<0.01; *, P<0.05). (**D**) B16-F10 cells transfected with either pLacIOP (mock) or pLacIOP-caveolin-1 (cav1) were treated or not with 1mM IPTG for 24 hours and total protein extracts were prepared, separated by SDS-PAGE (35 µg total protein per lane) and analyzed by Western Blotting. (**E**) Confluent monolayers of pLacIOP-caveolin-1 transfected B16-F10 cells in the absence or presence of 1mM IPTG were wounded with a pipet tip and fixed either at 0 or 60 minutes after wounding. Samples were stained with anti-caveolin-1 polyclonal antibody (green) and DAPI (blue). The edge of the wound is outlined with a white line. Scale bar, 20 µm. (**F**) Caveolin-1 localization was analyzed at different time points, as described in C. Data are the mean ± SEM from three independent experiments.

**Figure 2:**
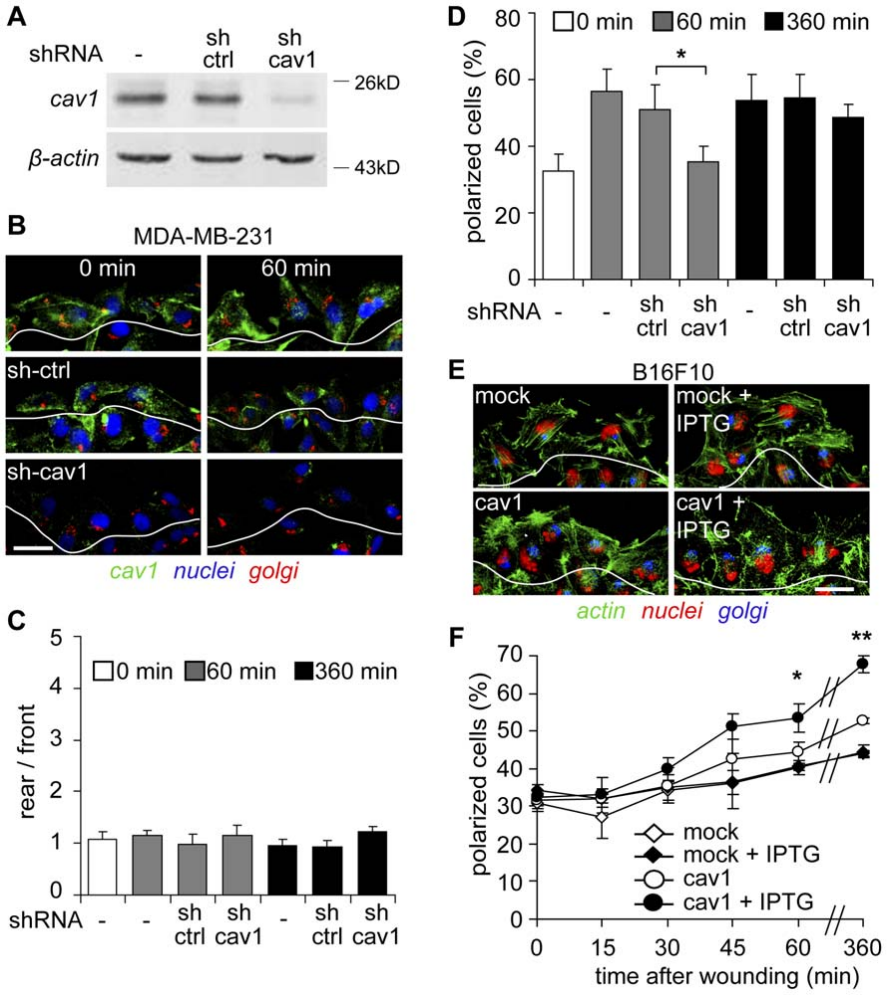
Polarization of metastatic cells is dependent on caveolin-1. (**A**) Total protein extracts were prepared from both parental MDA-MB-231 and cells stably transduced with a control shRNA targeting luciferase (sh-ctrl) or a shRNA targeting caveolin-1 (shRNA-caveolin-1 #5, sh-cav1). Extracts were separated by SDS-PAGE (35 µg total protein per lane) and analyzed by Western Blotting with antibodies against caveolin-1 and β-actin. (**B**) Confluent monolayers of parental, shRNA-control (sh-ctrl) or shRNA-caveolin-1 (sh-cav1) treated MDA-MB-231 cells were wounded with a pipet tip and fixed at 0 (left panel) and 60 minutes (right panel) after monolayer injury. Samples were stained with anti-caveolin-1 (green) and anti-Golgin-97 (red) antibodies, whereas the nuclei were stained with DAPI (blue). The first layer of cells facing the wounded area is distinguished from the rest by a white line. Scale bar, 20 µm. (**C**) Caveolin-1 localization was analyzed as described in Figure 1C, by measuring the ratio of fluorescence intensity “rear/front”. Data are the mean ± SEM, n = 3. (**D**) Polarization of both parental (-) and shRNA treated MDA-MB-231 cells with shRNA-luciferase (sh-ctrl) or shRNA-caveolin-1 (sh-cav1) was measured from (C) as described in the Materials and Methods, by analyzing the outer layer of cells facing the wounded area (B). The percentage of polarized cells was evaluated at 0, 60 and 360 minutes after wounding. Data were averaged from three independent experiments (mean ± SEM). *Comparison with control shRNA P<0.05. (**E**) B16-F10 cells transfected with either pLacIOP (mock) or pLacIOP-caveolin-1 (cav1) were treated or not with 1mM IPTG for 24 hours, grown in monolayers and wounded with a pipet tip to allow migration for 1 hours. Samples were stained with anti-Gigantin-1 polyclonal antibody (blue), phalloidin-Alexa488® (green) and propidium iodide (red). The outer layer of cells facing the wounded area is outlined with a white line. (**F**) Cell polarization was measured as described in the Materials and Methods, by analyzing the outer layer of cells facing the wounded area. The percentage of polarized cells was evaluated 0-360 minutes after wounding the monolayer. Data were averaged from three independent experiments (mean ± SEM). Statistically significant differences compared with pLacIOP-transfected B16-F10 (mock) cells are indicated (**, P<0.01 at time 360 min; *, P<0.05 at time 60 min).

Previous studies have shown that caveolin-1 accumulates at the rear of migrating cells including MEFs, HUVECs and neurons [19], [20], [21]. We sought to extend these observations to rapidly migrating metastatic cells, by assessing the localization of caveolin-1 in a wound-healing assay followed by immunofluorescence analysis. The human breast cancer cell line MDA-MB-231 and DI-TNC1, as well as MEF-3T3 cells, all expressed high endogenous levels of caveolin-1 (Figure 1A). Unexpectedly, caveolin-1 failed to accumulate at the rear of MDA-MB-231 cells during migration towards the wounded area (Figure 1B). In agreement with previous reports [8], [9], polarization of caveolin-1 was detected in MEF-3T3 fibroblast cells, as well as in astrocytic DI-TNC1 cells (Figure 1B). To evaluate the extent of caveolin-1 polarization, fluorescence intensities at the rear and front were quantified within discrete zones using the *Image J* software and the rear/front ratios were calculated at different time points of migration, as previously described [21], [23]. For both MEF-3T3 and DI-TNC1, but not MDA-MB-231 cells, time-dependent increases in caveolin-1 polarization were detected, whereby accumulation at the rear was almost complete after 360 minutes of migration (Figure 1C). Lack of caveolin-1 polarization in MDA-MB-231 cells could not be attributed to high endogenous expression levels in these cells, since, following shRNA-mediated down-regulation of caveolin-1, remnant caveolin-1 failed to polarize in these cells upon migration (see text below, Figures 2A, 2B and 2C).

To extend our findings in MDA-MB-231 cells, caveolin-1 polarization was also evaluated in mouse melanoma B16-F10 cells. These cells express low endogenous levels and, therefore, caveolin-1 was introduced by stably transfecting cells with the placIOP plasmid containing an insert encoding the full-length protein. An advantage of this plasmid is that it permits IPTG-inducible expression [26]. As shown, transfection with pLacIOP-caveolin-1 (*cav1 condition*) led to a 5-fold increase in caveolin-1 levels when compared with cells transfected with the empty vector pLacIOP (*mock condition*, Figure 1D). Expression was further increased with 1mM IPTG by another 5-fold when compared to non-induced cells (Figure 1D). Thus, caveolin-1 localization was evaluated in both induced (high expression) and non-induced cells (low expression) upon migration in a wound-healing assay. As for MDA-MB-231 cells, caveolin-1 failed to re-localize in migrating B16-F10 cells (Figure 1E) and accumulation at the rear was never observed at any time during migration (Figure 1F).

Taken together, these results suggest that, contrary to observations in non-metastatic cells evaluated here (MEF-3T3, DI-TNC1) and in the literature [19], [20], [21], caveolin-1 does not undergo polarization during migration of metastatic cells. Since the consequences of such caveolin-1 behavior for cell migration are unknown, the contribution of caveolin-1 to several migration-associated characteristics of MDA-MB-231 and B16-F10 cells was evaluated in the following experiments.

Cell polarization is the first step required for cell migration and directional movement. Re-localization of the MTOC ahead of the nuclei and behind the Golgi is important in defining the direction of migration [27]. To examine the role of caveolin-1 in MDA-MB-231 cell polarization, endogenous protein was down-regulated by shRNA (shRNA-caveolin-1 #5) (Figure 2A). As observed in Figure 1, residual endogenous caveolin-1 did not accumulate at the rear of migrating cells (Figures 2B, 2C). However, polarization of MDA-MB-231 cells with decreased caveolin-1 expression was substantially reduced, as shown by Golgi orientation (Figures 2B, 2D; Figure S1A, S1B). Quantitative analysis indicated that shRNA-mediated down-regulation of caveolin-1 delayed MDA-MB-231 cell polarization, since differences were no longer significant after 360 minutes (Figure 2D). As might be predicted for a dose-dependent caveolin-1 effect, partial loss of cell polarization was observed with another shRNA (shRNA-caveolin-1, sequence#3) that targeted caveolin-1 with lower efficiency (Figure S1A, Figure S1C). Thus, caveolin-1 appears to play an early, but transient role in the polarization of metastatic MDA-MB-231 cells. Likewise, B16-F10 cell polarization increased in a time-dependent fashion upon migration into a wounded area and the number of polarized cells was substantially higher in caveolin-1 expressing cells following IPTG induction (Figures 2E, 2F; Figure S2). A smaller increase in cell polarization was observed in low caveolin-1-expressing cells (no IPTG induction) when compared with the mock control (Figure 2F). These results indicate that in both B16-F10 mouse melanoma and MDA-MB-231 human breast cancer cells, caveolin-1 promotes cell polarization in a manner that does not require caveolin-1 accumulation at the cell rear.

### Caveolin-1 Promotes Migration of Metastatic Cells

**Figure 3:**
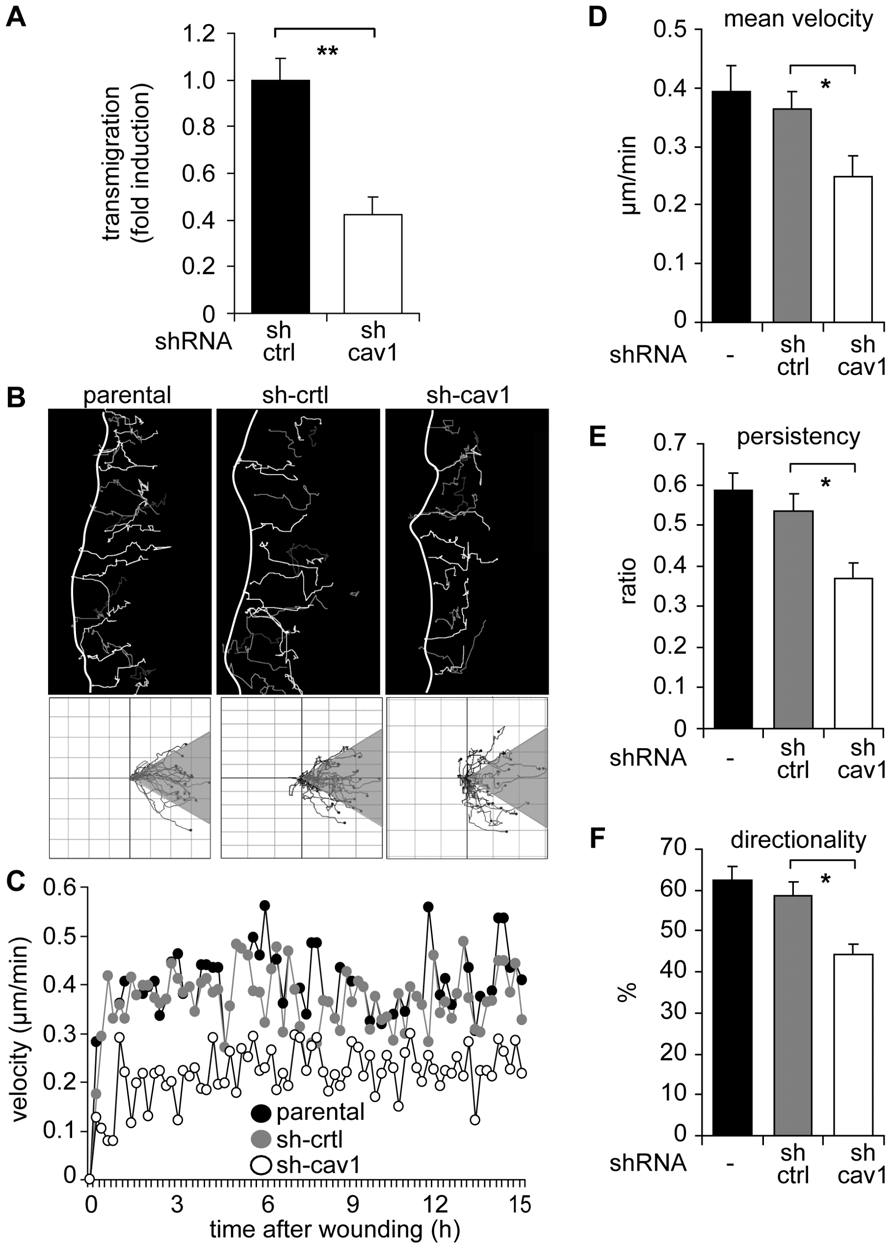
Caveolin-1 favors directionality and persistence of migration in MDA-MB-231 cells. (**A**) Migration of MDA-MB-231 cells treated with shRNA targeting either luciferase (sh-ctrl) or caveolin-1 (sh-cav1) was assessed in a Boyden chamber assay. Cells (5×10^4^) were seeded on fibronectin-coated (2 µg/ml) transwell plates and allowed to migrate for 2 hours. Cells that migrated to the lower side were detected with crystal violet staining (mean ± SEM, n = 3). **P<0.01. (**B**) Parental and shRNA treated MDA-MB-231 cells were grown as confluent monolayers, wounded with a pipet tip and migration was recorded by time-lapse video microscopy (total 15 hours, 12 minutes frame interval). Cell tracks were determined by using the *Image J* software (*“Manual Tracking”* plug-in). Tracks of single cells at the wounded edge are shown (upper panel). The edge of the wound is indicated with a white line. Single cell tracks are shown in a Cartesian coordinate system for each cell type (lower panel). (**C**) Instant velocity was analyzed for each cell type in (B) and plotted as a function of time (0-15 hours). (**D**) Mean velocity values were obtained from (C) and plotted for each cell type. (**E**) Persistency of migration was calculated as the ratio between the net distance and the total distance of migration in (B). Average values were obtained for parental, sh-ctrl and sh-cav1 cells (mean ± SEM). (**F**) Directionality of migration was obtained from the analysis shown in (B), lower panels. Tracks that lay within a 60° angle with respect to the direction of cell movement were considered as oriented (shaded region). The percentage of cells with orientated tracks was calculated and plotted (mean ± SEM, n = 3). *P<0.05.

**Figure 4:**
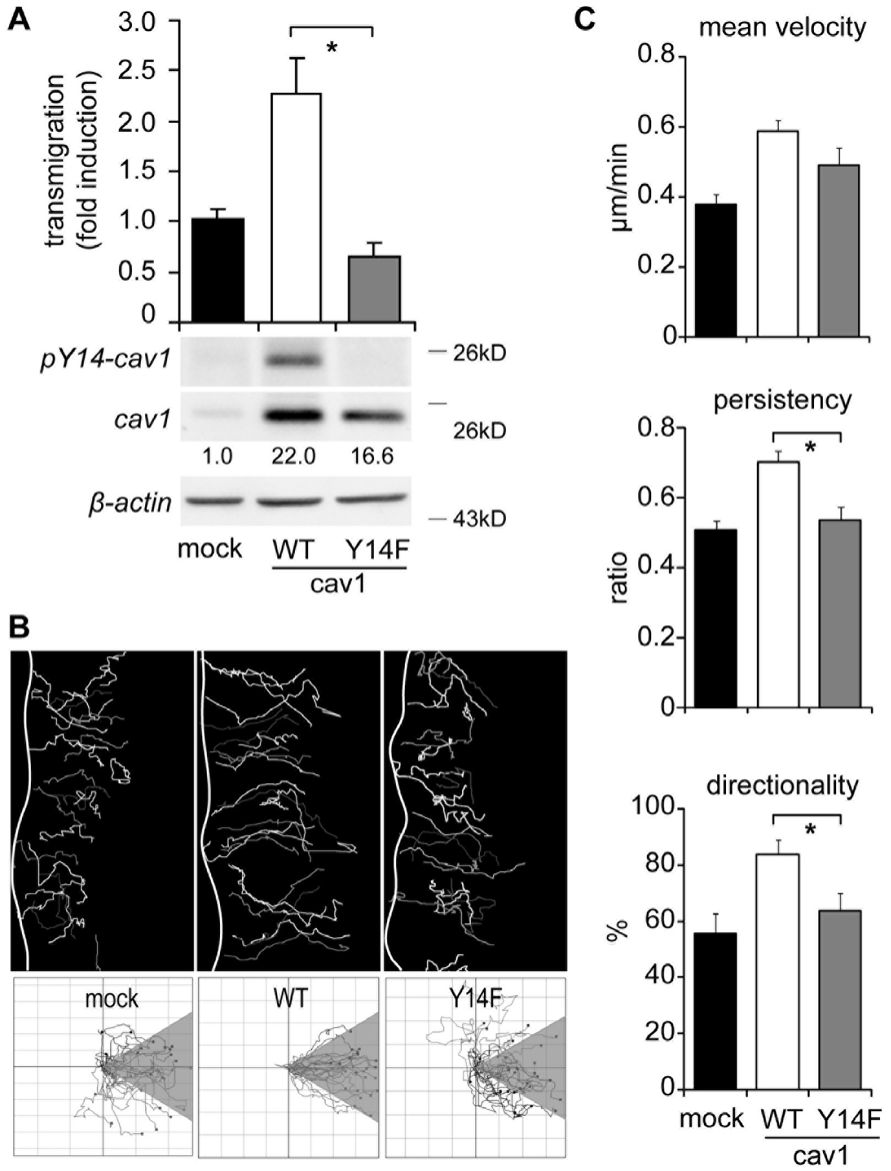
Caveolin-1 aminoacid tyrosine-14 is required for directionality and persistence of migration in B16-F10 cells. (**A**) B16-F10 cells transfected with pLacIOP (mock), pLacIOP-caveolin-1 (WT) or the pLacIOP-caveolin-1/Y14F mutant (Y14F) were treated with 1mM IPTG for 24 hours. Then, cell migration was assessed in a Boyden chamber assay by seeding cells (5×10^4^) on fibronectin-coated (2 µg/ml) transwell plates and allowing migration for 2 hours. Cells that migrated to the lower side were detected by crystal violet staining (mean ± SEM, n = 3). *P<0.05. Bottom panels show total protein extracts, separated by SDS-PAGE (35 µg total protein per lane) and analyzed by Western Blotting. Numbers below panels indicate relative caveolin-1 levels (caveolin-1/WT = 22.0±1.8, caveolin-1/Y14F = 16.6±3.8; values compared with the mock condition and obtained as the mean from three independent measurements ± SEM). (**B**) Confluent monolayers of B16-F10 cells transfected with pLacIOP (mock), pLacIOP-caveolin-1 (WT) or pLacIOP-caveolin-1/Y14F (Y14F) were wounded with a pipet tip and recorded by time-lapse video microscopy for 10 hours (12-min frame interval). Cell tracks were determined as indicated in Figure legend 3B. Tracks of single cells at the wounded edge are shown (upper panel). The edge of the wound is outlined by a white line. Single cell tracks are shown in a Cartesian coordinate system for each cell type (lower panel). (**C**) The mean velocity ( µm/min) was derived from the instant velocity (Figure S3), as described in (D). The persistency and directionality of migration were obtained as described in Figures 3E and 3F, respectively. Data shown are the mean ± SEM (n = 3). *P<0.05.

The role of caveolin-1 in cell migration is controversial and appears to be cell-context dependent, since it has been described both as a positive [18], [19] and negative regulator of migration [28], [29], [30]. In these studies predominantly fibroblasts and endothelial cells were characterized. Thus, we evaluated the effect of caveolin-1 in migration of metastatic MDA-MB-231 and B16-F10 cells. In both cases, caveolin-1 expression favored migration, as observed in a Boyden Chamber assay (Figures 3A, 4A) and upon analysis by time-lapse video microscopy combined with individual cell tracking (Figures 3B, 4B). Caveolin-1 down-regulation in MDA-MB-231 cells by shRNA decreased track length and directionality of migration when compared with parental and control cells (Figure 3B). The data support the idea that caveolin-1 presence in these cells favors persistence of cell migration. Indeed, caveolin-1 down-regulation decreased cell migration associated parameters, including instant (Figure 3C) and mean velocity (Figure 3D), persistency (obtained as the ratio between net and total distance, Figure 3E) and directionality of migration (expressed as the percentage of cells that move within a 60° angle from the starting point) (Figures 3B, 3F). Similar results were obtained in B16-F10 cells, where caveolin-1 expression increased transmigration in a Boyden Chamber assay (Figure 4A) and migration in a wound-healing assay (Figure 4B), as well as the instant and mean velocity, persistency and directionality of migration (Figure 4C, Figure S3). These data are in agreement with previous observations in MEF-3T3 cells [10], [11], [18] and suggest that caveolin-1 modulates the migration of metastatic MDA-MB-231 and B16-F10 cells, but does so even in the absence of changes in intracellular distribution.

### Tyrosine Phosphorylation on Caveolin-1 is Required for Migration of Metastatic Cells

Caveolin-1 undergoes Src-mediated phosphorylation on tyrosine-14, which is thought to be essential for fibroblast cell migration [18]. The involvement of phosphorylation of caveolin-1 on tyrosine-14 in promoting cell migration is further supported by observations in breast cancer cell lines, including MDA-MB-231 cells [11]. Thus, we evaluated the role of tyrosine phosphorylation in the B16-F10 melanoma cell line. Intriguingly, expression of non-phosphorylatable caveolin-1 (mutant Y14F) failed to promote B16-F10 cell migration (Figure 4A, 4B). Although the Y14F mutant was not expressed to the same extent as the wild type protein, it is unlikely that such differential expression accounts for inability of this protein to promote cell migration (Figure 4A), since for a population of B16F10(caveolin-1) cells (clone 3) with comparable levels of caveolin-1 to those detected in caveolin-1(Y14F) cells (see Figure S3), migration rates were similar to those detected for the original population of B16F10(caveolin-1) cells (Figure 4A). Video microscopy analysis showed that tracks of mock controls and caveolin-1(Y14F) cells were shorter than those obtained from cells expressing wild-type caveolin-1 (Figure 4B). Additionally, migration was more random in cells lacking caveolin-1 or expressing caveolin-1(Y14F) and parameters of mean velocity, persistency and directionality of migration were lower in caveolin-1(Y14F) cells when compared with cells expressing wild-type caveolin-1 (Figure 4C). These results indicate that caveolin-1 promotes cell migration in B16-F10 cells by increasing cell polarization, velocity, persistency and directionality of cell movement and that the ability to do so depends on the presence and presumably phosphorylation of tyrosine-14.

**Figure 5:**
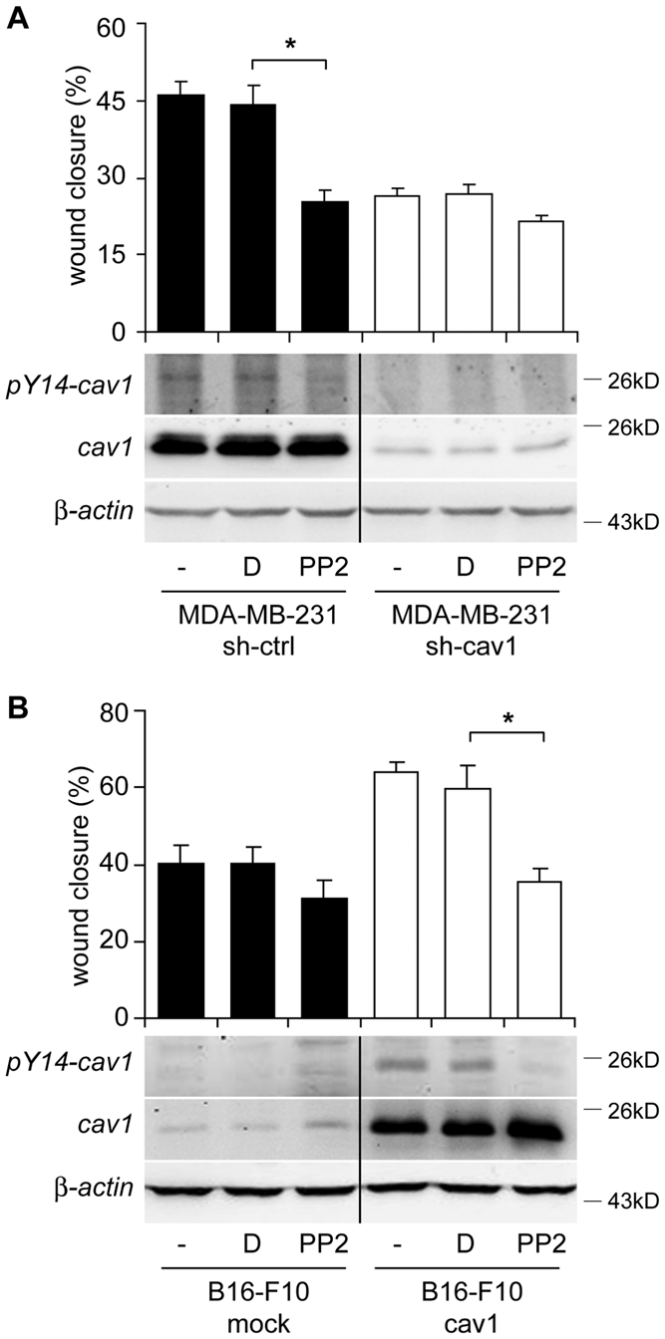
Inhibition of Src family kinases decreases cell migration and phosphorylation of caveolin-1 on tyrosine-14. (A) MDA-MB-231 cells stably transduced with shRNA either targeting Luciferase (sh-ctrl) or caveolin-1 (sh-cav1) were pre-incubated with DMSO (D) or 10 µM PP2 for 30 minutes, wounded with a pipet tip, stimulated (+) or not (-) with 3% of serum and images were recorded at 0 and 16 hours post-wounding. The wounded area was measured with the *Image J* software and the percentage of wound closure was plotted (upper panel). Total protein extracts were prepared and analyzed by Western blotting with antibodies against caveolin-1, pY14-caveolin-1 and actin. (**B**) Confluent monolayers of B16-F10 cells transfected with either pLacIOP or pLacIOP-caveolin-1 were pre-incubated with 1 mM IPTG for 24 hours. Then, cells were treated with DMSO (D) or PP2, wounded with a pipet tip, stimulated with 3% of serum and analyzed at 0 and 7 hours post-wounding, as described in (A). Total protein extracts were prepared and analyzed by Western blotting. Values in (A) and (B) were averaged from 3 independent experiments (mean ± SEM). *P<0.05.

Data obtained in B16-F10 cells were further validated by pharmacological inhibition of the Src family kinases, which phosphorylate caveolin-1 on tyrosine-14 [31]. Cell migration and phosphorylation of caveolin-1 were evaluated in the presence or absence of the selective Src kinase inhibitor, PP2 (4-amino-5-(4-chlorophenyl)-7-(dimethylethyl)pyrazolo[3,4-d]pyrimidine), in both MDA-MB-231 and B16-F10 cells. Serum stimulation increased wound closure in both MDA-MB-231 and B16-F10 cells. Wound closure was modulated by caveolin-1 expression, as shRNA mediated down-regulation in MDA-MB-231 cells decreased (Figure 5A, histogram bars), whereas ectopic expression in B16-F10 cells increased wound closure (Figure 5B, histogram bars). Importantly, treatment with PP2, but not control vehicle (dimethyl sulfoxide, DMSO) prevented caveolin-1-enhanced wound closure in both MDA-MB-231 (Figure 5A) and B16-F10 cells (Figure 5B). Moreover, both endogenous and ectopically expressed caveolin-1 failed to undergo phosphorylation in the presence of this inhibitor (Figures 5A, 5B, Western blots). It should be noted that little pY14-caveolin-1 was detectable in the mock-transfected B16-F10 cells, presumably because endogenous levels of caveolin-1 were too low. Importantly, no significant changes in proliferation were observed for both cell lines in presence of the inhibitor (data not shown). These results are consistent with the idea that the ability of caveolin-1 to enhance metastatic cell migration depends on tyrosine-14 phosphorylation by Src family kinases.

### Caveolin-1 Promotes Focal Adhesion Turnover in Metastatic Cells

**Figure 6:**
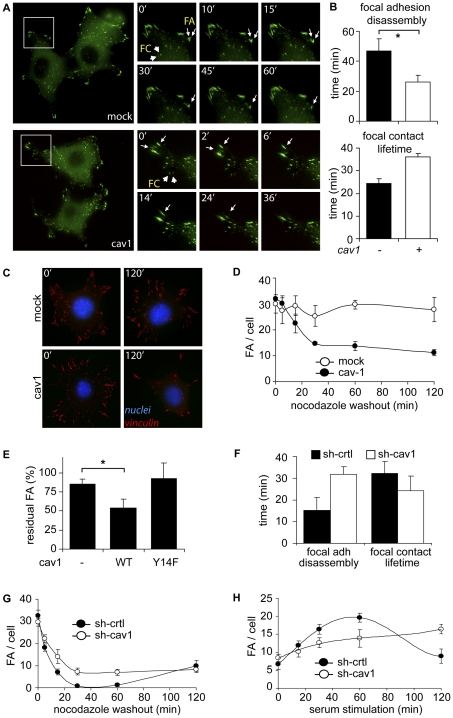
Caveolin-1 enhances focal adhesion turnover in metastatic cells. (**A**) B16-F10 cells transfected with pLacIOP (mock) or pLacIOP-caveolin-1 (cav1) were transfected with pEGFP-vinculin for 24 hours, seeded on fibronectin-coated coverslips (2 µg/ml) and grown in the presence of 1 mM IPTG for 24 hours (see Materials and Methods). Then, cells were serum-starved for 4 hours, pulsed with 10% serum and recorded by time-lapse video microscopy for 60 minutes (1-min frame interval). Zoomed areas are shown at selected times for mock and cav1 cells. Focal adhesions (FA, arrows) and focal contacts (FC, arrowheads) were defined by size. Images are representative of 3 independent experiments. (**B**) Kinetics of FAs and FCs were measured from experiments shown in A. FA disassembly and FC lifetime were measured for at least 6 structures per experiment (mean ± SEM, n = 3). *P<0.05. (**C**) B16-F10 mock or cav1 cells were seeded onto fibronectin-coated coverslips (2 µg/ml), grown in the presence of 1 mM IPTG for 24 hours and treated with 10 µM nocodazole in serum-free medium for 4 hours. Then, nocodazole was removed by wash-out with serum-free medium and cells were incubated by 0–20 minutes at 37°C. Subsequently, cells were fixed and stained with anti-vinculin antibody (red) and DAPI (blue) to label FAs and nuclei, respectively. (**D**) FAs were quantified from experiments described in (C) by using the *Image J* software. At least 10 images per time point were analyzed. Data were averaged from three independent experiments (mean ± SEM, n = 3). (**E**) B16-F10 mock (-), cav1 (WT) or cav1/Y14F (Y14F) cells were treated with 10 µM nocodazole, as described in Figure 6C. Nocodazole was subsequently washed-out for 60 minutes and FAs were analyzed with an anti-vinculin antibody (red) and nuclei with DAPI (blue). FAs were quantified as in (D). Data are representative from three independent experiments (mean ± SEM). *P<0.05. (**F**) MDA-MB-231 cells stably transduced with shRNA either targeting Luciferase (sh-ctrl) or caveolin-1 (sh-cav1) were transfected with pEGFP-vinculin for 24 hours, serum-starved for 4 hours, pulsed with 10% serum and recorded by time-lapse video microscopy, as described in (A). FA disassembly and FC lifetime were measured for 5 structures per experiment. Data were averaged from three independent experiments (mean ± SEM, n = 3). (**G**) Focal adhesion disassembly was measured after nocodazole removal in MDA-MB-231 cells treated with shRNA targeting luciferase (sh-ctrl) or caveolin-1 (sh-cav1), as described in (C) and (D). Data were averaged from 12 measurements (mean ± SD). Data are representative of two independent experiments. (**H**) FA assembly was evaluated as described in the Materials and Methods. MDA-MB-231 cells were treated with 10 µM nocodazole for 4 hours and subsequently washed-out for 45 min. Then, cells were pulsed with 10% serum for the indicated periods of time and FAs were evaluated as described in (D). Data were averaged from 12 measurements (mean ± SD). Data are representative of two independent experiments.

Caveolin-1 decreases the shuttling of FAK between focal adhesion and cytosolic pools, suggesting a role for caveolin-1 in the turnover of FAs [24]. Most importantly, cell migration is tightly regulated at the level of FA turnover [32], [33]. Thus, we evaluated the effect of caveolin-1 on FA turnover in metastatic cancer cells by transfection with GFP-vinculin followed by time-lapse videomicroscopy analysis. GFP-vinculin was associated with FAs of both mock and caveolin-1 expressing B16-F10 cells (Figure 6A). Although a small percentage of total FAs underwent turnover in B16-F10 cells (5–10% of total FAs), a significant increase in the kinetics of FA disassembly was observed in caveolin-1 expressing cells when compared with the mock control (Figure 6A, arrows; Figure 6B, top graph). Importantly, expression of caveolin-1 increased focal contact (FC) lifetime (Figure 6A, arrowheads; Figure 6B, bottom graph), in agreement with previous observations [18].

Alternatively, the effect of caveolin-1 on FA turnover was evaluated in nocodazole-treated cells. Disruption of microtubules with nocodazole is known to trigger enhanced/synchronized FA formation in a reversible manner [34]. Thus, B16-F10 cells were incubated with nocodazole in order to induce FA formation and the kinetics of FA disassembly was evaluated upon nocodazole removal. As shown, expression of caveolin-1 promoted FA disassembly after 120 minutes of nocodazole removal (Figure 6C) and significantly enhanced the kinetics of turnover when compared with mock cells (Figure 6D). Importantly, these events depended on the presence of tyrosine-14, as expression of non-phosphorylatable caveolin-1 (mutant Y14F) failed to enhance FA turnover after nocodazole removal (Figure 6E, Figure S4).

These observations were further evaluated in MDA-MB-231 cells. As anticipated, the time required for FA disassembly increased, whereas FC lifetime decreased upon down-regulation of caveolin-1 by shRNA (Figure 6F). Similarly, FAs disassembled in both shRNA-control and shRNA-caveolin-1 cells upon nocodazole removal; but a significant delay in FA disassembly was observed in cells treated with shRNA targeting caveolin-1 (Figure 6G). Indeed, both the parameters, half-life (t_1/2_ shRNA-control = 7 min; t_1/2_ shRNA-caveolin-1 = 10.5min) and initial velocity (V_0_ shRNA-control = -1.2 FA/min/cell; V_0_ shRNA-caveolin-1 = -0.6 FA/min/cell) were altered by caveolin-1 depletion in these cells.

Rather intriguingly, after extended periods following nocodazole removal (> 60 min), an increase in FA number was observed only in caveolin-1 expressing cells (shRNA-control), further suggesting that caveolin-1 is involved in FA assembly (Figure 6G). To test this hypothesis, FAs were allowed to accumulate in cells by treatment with nocodazole. Then the reagent was removed by changing the medium (wash out), which permits evaluating the maximum rate of FA disassembly (45 min after removal). Cells were then pulsed with serum for different periods of time, in order to induce FA formation, and FAs were quantified (more details in the Materials and Methods). As anticipated, caveolin-1 depletion delayed the kinetics of FA assembly (Figure 6H) in MDA-MB-231 cells. In addition, as for disassembly, both parameters of FA assembly, half-life (t_1/2_ shRNA-control = 17 min; t_1/2_ shRNA-caveolin-1 = 20 min) and initial velocity of assembly (V_0_ shRNA-control = 0.34 FA/min/cell; V_0_ shRNA-caveolin-1 = 0.21 FA/min/cell) were altered upon caveolin-1 depletion in these cells. Collectively, results obtained in MDA-MB-231 and B16-F10 cells indicate that caveolin-1 modulates FA dynamics, both at the level of assembly and disassembly. Additionally, caveolin-1 seems also to impact on FC stability, as shown in metastatic cancer cells (Figures 6B, F) and MEFs [18].

### Effect of Caveolin-1 on the Rho-GTPase Balance of Metastatic Cells

**Figure 7:**
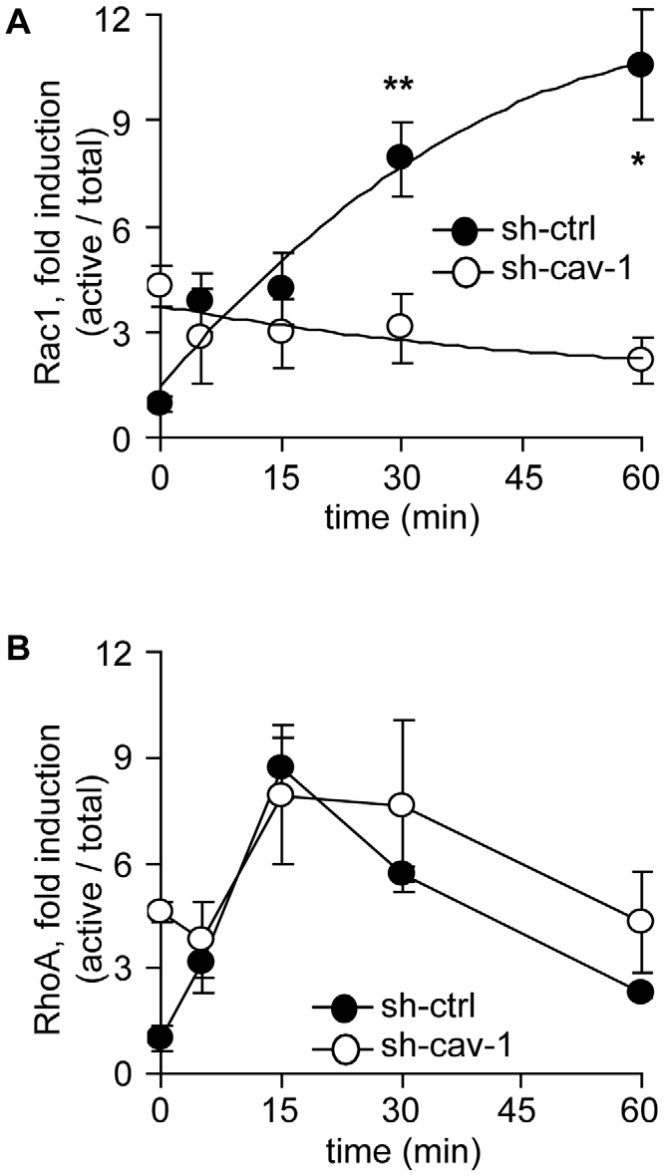
Effects of caveolin-1 on Rho-GTPases in MDA-MB-231 cells. (**A**) Kinetics of Rac1 activation upon spreading. MDA-MB-231 cells, stably transduced with shRNA either targeting Luciferase (sh-ctrl) or caveolin-1 (sh-cav1), were allowed to attach/spread onto fibronectin-coated plates (2 µg/ml) for different periods of time. Cell extracts were prepared and Rac1-GTP levels were measured by using the GST-PBD pull down assay, as indicated in the Materials and Methods. Rac1-GTP levels were normalized to total Rac1 by scanning densitometry. Data were averaged from three independent experiments (mean ± SEM). *P<0.01; **P<0.05. (**B**) RhoA activity was measured by the GST-RBD pull down assay in cells induced to spread onto fibronectin at different time points, as in (A). Data were averaged from three independent experiments (mean ± SEM).

Caveolin-1 was previously suggested to promote basal RhoA activation, while decreasing active Rac1 in fibroblasts. This regulation is thought to be essential for nascent FA stabilization by providing traction forces [18]. Coordinated activation of both GTPases is proposed to be essential during cell migration [3], [35]. Therefore, we evaluated the kinetics of activation of both RhoA and Rac1 during cell adhesion and spreading, two early events associated with cell migration. For these experiments, cells were seeded on plates covered with the extracellular matrix protein fibronectin, which favors adhesion in comparison to migration. It should be noted that expression of caveolin-1 did not affect cell adhesion to fibronectin in either MDA-MB-231 or B16-F10 cells (Figure S5). In accordance with previous findings, depletion of caveolin-1 increased basal Rac1 activity in metastatic MDA-MB-231 cells, as shown by comparing shRNA-control versus shRNA-caveolin-1 cells (Figure 7A, time = 0 min). Importantly, cell spreading induced Rac1 activation in a caveolin-1-dependent manner, since shRNA targeting abolished any further Rac1 activation (Figure 7A). Moreover, RhoA activation was induced both in cells expressing or lacking caveolin-1 and no significant differences were observed during cell spreading (Figure 7B).

## Discussion

The role of caveolin-1 in cell migration is controversial. On the one hand, evidence is available indicating that caveolin-1 promotes migration in a variety of cells including fibroblasts, endothelial cells and tumor-derived cell lines [11], [18], [19]. Alternatively, inhibition of migration is observed for astrocytes, endothelial, pancreatic carcinoma and metastatic breast cancer cells [28], [29], [30], [36]. In metastatic breast adenocarcinoma cells, caveolin-1 presence is associated with reduced extension of cellular protrusions and migration following stimulation with EGF [30]. Moreover, a recent report suggests that caveolin-1 expression in B16-F10 mouse melanoma cells decreases cell migration and experimental metastasis [37]. Alternatively, augmented caveolin-1 expression has been associated with enhanced metastatic potential and migration of lung and prostate cancer cells [38], [39]. Often, the ability to enhance migration correlates with polarization and accumulation of the protein at the rear end of migrating cells [3]. As a first step towards characterizing better the role of caveolin-1 in metastasis, we evaluated the impact of expression of this protein on the migration of two metastatic cell lines of different origin. Our findings in both human breast cancer (MDA-MB-231) and mouse melanoma (B16-F10) cells support the notion that caveolin-1 enhances polarization, directional migration and persistency of metastatic cells. However, rather surprisingly, this was achieved without any apparent redistribution or accumulation at the cell rear, although phosphorylation on tyrosine-14 appeared essential.

Previous reports showed that caveolin-1 redistribution and localization at the rear of migrating cells is essential for fibroblast migration [21], [23]. In migrating endothelial cells, caveolin-1 co-localizes with Gαq_/11_ at sites of calcium entry implicated in calpain activation required for FA disassembly and rear end retraction of cells [9]. Alternatively, some studies have suggested that the pool of caveolin-1 relevant for migration is phosphorylated on tyrosine-14 and located at the leading edge of migrating cells [19]. The interpretation of the latter studies though, is hampered by the observation that the anti-phospho-caveolin-1 antibody employed recognizes a different protein associated with FAs in localization experiments [25]. However, mutational analysis (Y14F mutation of caveolin-1) revealed that phosphorylatable Y14 is responsible for caveolin-1 localization within structures required for migration, such as lamellipodia and invadopodia [11]. In any case, our studies analyzing bulk caveolin-1 distribution suggest that neither polarization nor accumulation of caveolin-1 at the rear end or the leading edge is required in migrating metastatic cells.

This inability to detect protein re-distribution could be attributed to high expression levels of caveolin-1 in the cells analyzed. However, we consider this an unlikely possibility for at least two reasons. First, caveolin-1 levels in the two metastatic model cell lines employed were comparable to those detected in NIH3T3 fibroblasts and DITNC1 astrocytes, where we did see changes in caveolin-1 distribution upon migration (see Figure 1). Second, we also did not see any degree of polarization in caveolin-1 distribution for the non-IPTG induced B16-F10(cav-1) cells or even the B16-F10(mock) cells, which have very low endogenous levels of expression. Likewise, in the MDA-MB-231 cells, where endogenous caveolin-1 expression was reduced using a shRNA approach, no polarization of the residual protein was detectable. Despite this evidence, we cannot completely exclude the possibility that redistribution of a minor, functionally relevant pool may have gone undetected, in our studies. Alternatively, understanding how a key molecule like caveolin-1 participates in a process that requires polarization without itself being or becoming polarized is an enigma that will require further studies to be resolved.

Caveolin-1 effects on metastatic cell migration (this study) are reminiscent of those observed in fibroblasts [18]. Conversely, recent findings by Trimmer et al [37] suggest that caveolin-1 expression in B16-F10 mouse melanoma cells reduces migration. These discrepancies may be attributable to the experimental approaches used in the two studies. For instance, in this study we evaluated intrinsic cell migration rather than chemotaxis. Also, different approaches were employed to overexpress caveolin-1 in B16-F10 cells. The possibility that our results might be attributable to the nature of the expression system in B16-F10 cells can be ruled out as caveolin-1 dependent effects were observed in both low and high caveolin-1 expressing cells (IPTG absence or presence). Additionally, the validity of our results in B16F10 cells was confirmed by the observations in MDA-MB-231 cells where knock-down of endogenous caveolin-1 using shRNA lead to essentially the same conclusions.

Using MEFs from knock-out mice, caveolin-1 is shown to be essential for morphology, polarization and directional migration of these cells. The ability to do so in these cells is associated with changes in the activity of small G-protein family members. Thus, fibroblasts from knock-out mice reportedly have reduced Rho, but elevated Rac and Cdc42 activity. Indeed, caveolin-1 has been shown to regulate Src/p120rhoGAP and it has been suggested that this mechanism might contribute to caveolin-1-mediated control of small GTPase activity [18]. Besides the differences concerning polarization, others are apparent when comparing our results with those observed in fibroblasts models. First, caveolin-1 down-regulation in MDA-MB-231 cells did not affect significantly the kinetics of RhoA activation, whereas rather strikingly, Rac1-GTP loading increased upon attachment and cell spreading. Possibly, variations in the velocity of migration of these cells might be linked to such differences, given that MEF-3T3 fibroblasts and DI-TNC1 cells migrate slowly, while both MDA-MB-231 and B16-F10 cells are rapidly migrating cells. Corroborating this intriguing possibility will, however, require further research.

Alternatively, it should be mentioned that the observed differences may also be attributed to experimental settings. Previously reported data suggesting that Rho-GTPase activity increases in the presence of caveolin-1 were obtained with cells maintained in culture [18]. In our studies, on the other hand, we were interested in understanding the role of caveolin-1 in the initial stages of cell migration. Thus, enhanced caveolin-1-mediated activation of Rho-GTPases observed here may reflect the fact that cells freshly spreading on fibronectin were evaluated. In this context, it is important to note that cell adhesion to extracellular matrix proteins (i.e. fibronectin) did not depend on caveolin-1 expression in either MDA-MB-231 or B16-F10 cells, excluding the possibility that changes in migration were associated with differences in adhesion (Figure S5).

Another possibility that may help explaining apparent differences between previous reports and this study relates to the dynamics of FA turnover. Rapidly migrating cells assemble very transient FAs, whereas in slowly migrating cells FAs are larger and more stable. Such FAs are also a hallmark of adherent fibroblasts. With this in mind, we predicted that both MDA-MB-231 and B16-F10 cells should form relatively transient FAs that turnover even faster in the presence of caveolin-1. Indeed, caveolin-1 increased the kinetics of FA assembly and disassembly in both cell lines. FAK is widely considered a key regulator of FA dynamics [33]. Thus, our findings are in general agreement with data showing changes in FAK dynamics at FAs upon caveolin-1 expression [24].

Increased stability of FCs has been documented for fibroblasts upon caveolin-1 expression [18]. For both MDA-MB-231 and B16-F10 cells, a similar, but less pronounced increase in FC lifetime was linked to caveolin-1 expression. Again, these variations might be attributable to intrinsic cell properties, such as their speed of migration. Thus, in future studies, it will be interesting to compare both at the morphological and molecular level these FA structures in slowly and rapidly migrating cells.

Caveolin-1 is known to be phosphorylated by Src, Fyn (SFKs) or Abl under stress conditions, for example, following exposure of cells to H_2_O_2_ [40]. SFKs are also well-known regulators of cell migration [1], [3] and their inhibition reportedly affects FA turnover. Moreover, previous reports employing pharmacological and mutational approaches suggest that the phosphorylation of tyrosine-14 is essential for caveolin-1-mediated control of cell polarization, migration and FA dynamics [11], [17], [24]. Consistent with these published results, mutation of tyrosine-14 (Y14F mutant) ablated the ability of caveolin-1 to enhance FA turnover and to promote migration of B16-F10 cells. Alternatively, pharmacological inhibition of SFKs with the inhibitor PP2 in MDA-MB-231 cells coincided with reduced migration and the loss of caveolin-1 phosphorylation on tyrosine-14, again suggesting the requirement of this residue and phosphorylation thereof for caveolin-1-driven migration.

Caveolin-1 phosphorylation was previously associated with FA turnover by indirect measurements that assessed FAK stabilization at FAs [24], [41]. In addition, fluorescence recovery after photobleaching analysis suggested that FAK and FA regulation by caveolin-1 represent part of a feedback loop that involves Rho, ROCK, and Src [11]. In doing so, caveolin-1 was suggested to promote directional migration and changes in the Rho GTPase balance. Our results coincide in this respect by implicating SFK-dependent caveolin-1 phosphorylation in FA turnover in metastatic cells.

Caveolin-1 phosphorylation is implicated in promoting the translocation of Csk to caveolae and subsequent inactivation of Src and actin reorganization that could be related to the role of phospho-caveolin-1 in FA turnover and directional cell migration [31], [42]. Our results suggest that SFK activity is necessary for caveolin-1 phosphorylation and cell migration, but also that SFKs affect cell migration in a caveolin-1-independent manner, since cells with decreased caveolin-1 expression [shRNA-caveolin-1 in MDA-MB-231 cells and B16-F10 (mock) cells] showed a basal migration rate that was sensitive to PP2. Given that PP2 is a selective rather than a specific inhibitor, the identity of the specific SFK involved in caveolin-1 phosphorylation during metastatic cell migration remains to be established.

Finally, caveolin-1 expression has been associated with metastasis of tumor cells, including prostate, breast and colon carcinomas. A plethora of mechanisms have been proposed, such as autocrine/paracrine regulation, elevated angiogenesis and multi-drug resistance amongst others [14], [15]. Indeed, some metastatic features have also been linked to caveolin-1, as its expression increases invasion and the biogenesis of invadopodia [13], and regulates MT1-MMP function within these structures [43]. In this regard, more experiments are required to assess the requirement of this critical tyrosine-14 for caveolin-1-enhanced metastasis. Indeed, preliminary data suggest that mutation of this residue to phenylalanine compromises caveolin-1-enhanced metastasis of B16-F10 cells to the lungs of C57/BL6 mice (Lobos and Quest, data not shown). Thus, caveolin-1-enhanced migration described here for metastatic cells *in vitro* appears to correlate well with their behavior anticipated *in vivo*.

In summary, we show here that caveolin-1 expression enhances velocity, directionality and persistency of migration of metastatic cancer cells and that this effect depends on the presence of tyrosine-14. Unexpectedly, and in striking contrast to previous reports, polarization of caveolin-1 did not appear to be necessary. Since two metastatic (breast and melanoma) cell types from different species (human and mouse) were characterized in a variety of settings, our observations have broad implications for the role of caveolin-1 in rapidly migrating cells and cancer.

## Materials and Methods

### Antibodies and Reagents

Rabbit polyclonal antibodies anti-caveolin-1 (Transduction Laboratories, Lexington, KY), anti-actin (R&D Systems, Minneapolis, MN) and anti-Gigantin-1 (Covance, Princeton, NJ), as well as the mouse monoclonal antibodies anti-pY14-caveolin-1 (Transduction Laboratories, Lexington, KY), anti-caveolin-1 (Transduction Laboratories, Lexington, KY) and anti-Golgin97 (Molecular probes, Eugene, OR) were used as indicated by the manufacturers. Goat anti-rabbit and goat anti-mouse IgG antibodies coupled to horseradish peroxidase (HRP) were from Bio-Rad Laboratories (Hercules, CA) and Sigma-Aldrich (St. Louis, MO), respectively. Alexa Fluor 488 goat anti-mouse IgG and Alexa Fluor 546 goat anti-rabbit IgG were from Molecular Probes. DAPI was from Sigma-Aldrich. Cy3-coupled goat anti-mouse, fluorescein isothiocyanate-coupled goat anti-rabbit and Cy5-coupled goat anti-rabbit IgG were from Jackson Immunoresearch (West Grove, PA). The EZ-ECL chemiluminescent substrate was from Biological Industries (Kibbutz Beit Haemek, Israel). The BCA protein determination kit was from Pierce (Rockford, IL). The Plasmid Midi Kit was from Qiagen (Valencia, CA). Hygromycin was from Calbiochem (La Jolla, CA). Fetal bovine serum (FBS) was from Biological Industries and Hyclone. Cell culture media and antibiotics were from GIBCO (Invitrogen). PP2 was from Enzo Life Science International (Plymouth Meeting, PA). All other reagents were from Sigma-Aldrich or of the highest grade available.

### Cell Culture

The rat astrocytic cell line DI-TNC1 (ATCC, #CRL-2005) was maintained in RPMI 1640 medium containing 5% FBS (Fetal Bovine Serum), 0.1 mM 2-mercaptoethanol and antibiotics (100 U/mL penicillin and 100 µg/mL streptomycin). Human breast cancer cells MDA-MB-231 (ATCC, #HTB-26) were maintained in DMEM-F12 medium containing 10% FBS and antibiotics. Mouse embryonic fibroblasts MEF-3T3 (ATCC, #CRL-2752) were maintained in DMEM medium supplemented with 10% FBS and antibiotics. Metastatic murine melanoma cells B16-F10 (ATCC, #CRL6475) were maintained in RPMI 1640 medium supplemented with 10% FBS and antibiotics. Human embryonic kidney 293T cells (HEK293T, ATCC, #CRL-11268) were maintained in DMEM high glucose supplemented with 10% FBS and antibiotics. Cells were cultured at 37°C and 5% CO_2_.

### Transfection of B16-F10 Melanoma Cells

The plasmids pLacIOP and pLacIOP-caveolin-1 were previously described [26]. B16-F10 cells were grown to 60-80% confluence in 10-cm plates and then electroporated with 25 µg of pLacIOP or pLacIOP-caveolin-1 plasmids in RPMI medium without serum using a pulse of 270 V and 1,600 µF in a cell Porator (GIBCO, Invitrogen). After electroporation, cells were plated in complete RPMI medium containing hygromycin (750 µg/mL) for 2 to 3 weeks to yield stably transfected B16-F10(mock) and B16-F10(Cav-1) cells, respectively.

### Lentiviral shRNA, Constructs and Stable Knockdown of Caveolin-1

The oligonucleotide containing shRNA candidates for caveolin-1 (#1-1, CCAGTTAGATTTAGGGATTTA; #1-2, CCGCTTGTTGTCTACGATCTT; #1-3, CGACGTGGTCAAGATTGACTT; #1-4, TGAAGCTATTGGCAAGATATT; and #1-5, GCTTCCTGATTGAGATTCAGT) or control shRNA for Luciferase (CGCTGAGTACTTCGAAATGTC) were cloned into the lentiviral vector pLKO.1, which contains a puromycin resistance gene and the U6 promoter to control the shRNA. Lentiviral particles were prepared by co-transfecting HEK-293T cells (packaging) with the pLKO.1 vector and plasmids encoding the envelope protein VSV-g (pHCMV-G) and the packaging plasmid p?8.9 (pCMV?R8.9) as described [44]. Post-transfection (48 hours), media containing lentivirus were filtered through a 0.45 µm filter and used to transduce MDA-MB-231 cells in the presence of 8 µg/ml polybreen. After 24 hours cells were selected with puromycin (2µg/ml) for seven days and expression was monitored by Western blotting. Plasmids encoding the envelope protein VSV-g (pHCMV-G), the packaging plasmid p?8.9 (pCMV?R8.9) and pLKO.1 plasmids containing shRNA for caveolin-1 and control plasmid containing shRNA for Luciferase (shLuc) were provided by Dr. Claudio Hetz (Universidad de Chile, Santiago, Chile).

### Site Directed Mutagenesis of Caveolin-1

The Y14F mutation was introduced by double PCR, using the primers 5′ - cct ctt tac cgt tcc cat cc - 3′ (sense) and 5′ - gaa cgg taa aga ggt gcc c - 3′ with sequence overlap in the region encompassing the codon for tyrosine 14. External primers used to amplify the full-length caveolin-1 sequence were: 5′-ccg agc gcg gcc gcc atg tct ggg ggc aaa tac-3′ (sense) and 5′-tat ctg gcg gcc gct tat gtt tct ttc tgc atg ttg-3′ (anti-sense), both harboring *Not*I sites. After a double PCR reaction, the final PCR product was cloned into pcDNA3.1(+) following digestion with a *Bam*HI/*Eco*RI. Positive colonies were identified and sequenced in both directions. The caveolin-1-encoding sequence with the Y14F mutation was then sub-cloned from pcDNA3.1(+) into the multiple cloning site of pLacIOP, by digesting with *Not*I. Correct orientation of the insert was determined by PCR using an external anti-sense primer targeting the vector (5′-gat gaa gaa ttc tta tgt ttc ttt ctg cat gtt g-3′) in combination with the sense primer used to generate the Y14F mutation.

### Western Blotting

Cells grown to 80% confluence were washed twice with cold PBS and lysed in 0.2 mM HEPES (pH 7.4) buffer containing 0.1% SDS, phosphatase inhibitors (1 mM Na_3_VO_4_) as well as a protease inhibitors cocktail (10 µg/mL benzamidine, 2 µg/mL antipain, 1 µg/mL leupeptin, 1 mM PMSF). Protein concentration was determined by the BCA assay. Total protein extracts (30 µg/lane) were separated by SDS-polyacrylamide gel electrophoresis (SDS-PAGE). Separated proteins were then transferred to nitrocellulose membrane. Blots were blocked with 5% milk in 0.1% Tween-PBS and then probed with anti-actin (1∶5000), anti-caveolin-1 (1∶5000) polyclonal antibodies or blocked with 5% Gelatin in 0.1% Tween-PBS for incubations with anti-pY14-caveolin-1 (1∶3000) monoclonal antibody. Bound antibodies were detected with HRP-conjugated secondary antibodies and the EZ-ECL system.

### Immunofluorescence Labeling and Cell Polarization Assay

Confluent cell monolayers grown on 12-mm coverslips in 24-well plates were injured with a 20-200 µl pipette tip, washed twice with PBS and subsequently RPMI 1640 with 3% FBS was added. Cells were fixed at different time points with 4% paraformaldehyde in 100 mM PIPES buffer pH 6.8, containing 40 mM KOH, 2 mM EGTA and 2 mM MgCl_2_ for 30 minutes. Afterwards, cells were washed three times with washing solution (50 mM Tris buffer pH 7.6 containing 0.15N NaCl and 0.1% sodium azide). Cells were permeabilized with 0.1% Triton X-100 in washing solution for 10 min, washed twice and then blocked with 1% bovine serum albumin for 60 min. Caveolin-1 polarization was evaluated by staining cells with an anti-caveolin-1 pAb (1∶200), followed by FITC-conjugated anti-rabbit IgG (1∶200). Cell polarization was monitored by staining with anti-Golgin97 mAb and anti-caveolin-1 pAb followed by rhodamine-coupled anti-mouse IgG and FITC-coupled anti-rabbit IgG for MDA-MB-231 cells, and anti-Gigantin-1 pAb followed by Cy5-coupled anti-rabbit IgG for B16-F10 cells. FITC-coupled phalloidin was used to stain polymerized actin. Either DAPI (0.5 µg/mL) or Propidium Iodide (10 µg/mL) were used for nuclear staining. Coverslips were washed and mounted on microscope slides with 10% Mowiol-2.5% 1,4-Diazabicyclo [2.2.2]octane and samples were visualized with a LSM Microsystems Pascal 5 confocal microscope (Carl Zeiss, Thornwood, NY).

Caveolin-1 polarization was evaluated in the first layer of cells lining the wounded area by scoring for Golgi orientation towards the wound. Eight equal regions were defined within each cell analyzed, four regions at the cell front and four at the rear, with respect to the nucleus. Fluorescence intensity was measured with the *Image J* software and the ratio of rear-to-front fluorescence intensity was calculated [21]. Cells harboring polarized caveolin-1 were defined as cells with a fluorescence intensity ratio 2 fold S.D. greater than the mean at time 0 min. Cell polarization was evaluated as the percentage of cells along the border of the wound that show reoriented Golgi with respect to the nuclei. Cells were considered polarized, when the Golgi was perinuclear and oriented towards the wounded area.

### Time-Lapse Video Microscopy

For cell migration track analysis, confluent monolayers were wounded with a 20-200 µl pipette tip. Cells were washed twice with PBS and subsequently RPMI 1640 with 3% FBS was added. Image series were acquired using a 10X objective lens in an inverted microscope (Leica TCS SP) heated with an airstream incubator at 37°C. Images were captured using a CCD Hamamatsu camera. Image processing and analysis was performed with the *Image J* software (Plugin “Manual Tracking”).

The velocity of migration was measured as the instant velocity of each cell at any given time point. Cell persistency was quantified as the ratio of the net distance divided by the total distance of movement (ID) for each cell. Directionality of cell migration (cell orientation) was evaluated with the *Image J* Software (plugin “chemotaxis”) by placing cell tracks in a Cartesian coordinate system. Cell tracks that remained within a 60° angle with respect to the direction of cell movement were considered as directional.

Vinculin is an intermediate filament protein that is recruited to FAs as they form and then degraded as these structures disassemble [45]. Thus, to evaluate FA turnover, cells were transiently transfected with plasmid encoding vinculin-GFP (pEGFP-vinculin, kindly donated by Kris DeMali, University of Iowa [46]). Post-transfection (24 hours), cells were re-plated onto 22 mm coverslips, grown for 24 hours in complete medium followed by 4 hours starvation and then stimulated (pulsed) with 10% FBS. Cells were visualized in a spinning disk confocal microscope (IX81, Olympus) and a 12-bit CCD camera (XM10, Olympus). Images were captured at time intervals of 1 minute for 1 hour. For the analysis of FAs and FCs, we first defined these structures in function of size with the *Image J* software. GFP-positive structures ranging from 20 to 850 relative pixels were screened and the cut-off was made as follows: 20-100 relative pixels were referred to as focal contacts (FCs) and 200-850 relative pixels were referred to as FAs (average relative pixels were 49 and 388 for FCs and FAs, respectively). FA turnover and half life was evaluated from time-lapse image series, by quantifying the intensity of pixels of nascent focal adhesions (focal contacts, FCs) and mature focal adhesions (FAs) with the *Image J* software.

### Focal Adhesion Assembly and Disassembly Assay

Cells were grown on glass coverslips for 24 hours and then treated with 10 µM nocodazole in serum-free medium for 4 hours to depolymerize microtubules, as previously described [34]. Nocodazole was washed out with serum-free medium and cells were incubated at 37°C for the indicated periods of time. Subsequently, cells were fixed and then prepared for immunofluorescence staining, as indicated above. FAs were identified with anti-vinculin antibody; actin was stained with phalloidin and nuclei with DAPI. Samples were visualized using spinning disk confocal microscopy (IX81, Olympus) and a 12-BIT CCD camera (XM10, Olympus). Images were processed after subtracting threshold levels due to diffuse vinculin staining. For evaluation of assembly and disassembly, FAs were quantified at all time points (total number). Fluorescence pixels due to vinculin accumulated in punctate structures was normalized with respect to the number of cells analyzed.

### Transwell Migration Assay

Assays were performed in Boyden Chambers (Transwell Costar, 6.5 mm diameter, 8 µm pore size) according to the manufacturer’s instructions. Briefly, the bottom sides of the inserts were coated with 2 µg/ml fibronectin. Cells (5×10^4^) re-suspended in serum-free medium were plated onto the top of each chamber insert and serum-free medium was added to the bottom chamber. After 2 hours, inserts were removed, washed and cells that migrated to the bottom side of the inserts were stained with 0.1% crystal violet in 2% ethanol and counted in an inverted microscope.

### Adhesion Assay

Cells were suspended in serum-free medium and allowed to attach to fibronectin coated-24 well plates (2µg/ml) at different periods of time as indicated. Non-adherent cells were removed by washing gently in serum-free medium and adherent cells were stained with 0.1% crystal violet in 2% ethanol. Cell-bound dye was eluted with methanol, and the absorbance was measured at 600 nm.

### Rac-GTP and RhoA-GTP Pull Down Assay

Cells were allowed to attach to fibronectin coated plates (2 µg/ml) for different periods of time and subsequently lysed in a buffer containing 25 mM HEPES (pH 7.4), 100 mM NaCl, 5 mM MgCl2, 1% NP40, 10% glycerol, 1 mM dithiothreitol and protease inhibitors. Extracts were incubated for 5 minutes on ice and clarified by centrifugation (10,000×g, 1 min, 4°C). Post-nuclear supernatants were used for pull down assays with 30 µg of GST-PBD (Rac1) or GST-RBD (RhoA) pre-coated GSH beads per condition. Beads were incubated with supernatant for 15 minutes at 4°C in a rotating shaker. Thereafter, beads were collected, washed with lysis buffer containing 0.01% NP40 and samples were solubilized in sample buffer, boiled and separated by SDS PAGE for analysis by Western blotting as indicated above.

### Statistical Analysis

Results were statistically compared using the Kruskal-Wallis ANOVA test followed by multiple comparison posttests (Dunn’s multiple comparison test). For paired groups the Mann-Withney test was employed. Data analyzed in this manner are specifically indicated in the respective Figure legends. All groups were from three or more independent experiments. *P<0.05* was considered significant.

## Supporting Information

Figure S1 **MDA-MB-231 cell polarization.** (A) Total protein extracts were prepared from parental MDA-MB-231 and cells stably transfected with a control shRNA targeting luciferase (sh-ctrl) or different shRNA sequences targeting caveolin-1 (sh cav1, #1-3 or #1-5). Extracts were separated by SDS-PAGE (35 µg total protein per lane) and analyzed by Western Blotting with antibodies against caveolin-1 and β actin. (B) Confluent monolayers of parental, shRNA-control (sh-ctrl) or shRNA-caveolin-1 (sh-cav1, #1-5) treated MDA-MB-231 cells were wounded with a pipet tip and fixed at 0 (left panel) and 60 min (right panel) after monolayer injury. Samples were stained with anti-Golgin-97 (red) antibody and the nuclei were stained with DAPI (blue). The edge of the wound is outlined by a white line. Scale bar, 20 µm. (C) Confluent monolayers of shRNA-caveolin-1 (sh-cav1, #1-3) treated MDA-MB-231 cells were analyzed as in (A) and stained with anti-Golgin-97 (red) antibody and the nuclei were stained with DAPI (blue).
(TIF)

Figure S2 **B16-F10 cell polarization.** B16-F10 cells transfected with either pLacIOP (mock) or pLacIOP-caveolin-1 (cav1) were treated or not with 1 mM IPTG for 24 hours, grown in monolayers and wounded with a pipet tip to allow migration for 1 hour. Samples were stained with anti-Gigantin-1 polyclonal antibody (blue) and propidium iodide (red). The edge of the wound is outlined by a white line. Scale bar, 20 µm.
(TIF)

Figure S3 **Effect of caveolin-1 on B16-F10 cell migration.** (**A**) B16-F10 cells transfected with pLacIOP (mock, open triangles), pLacIOP-caveolin-1 (WT, filled diamonds) or the pLacIOP-caveolin-1/Y14F mutant (Y14F, gray circles) were treated with 1 mM IPTG, grown as confluent monolayers and wounded with a pipette tip. Migration was recorded by time lapse video microscopy (total hours, 12 min frame interval) and cell tracks were determined by using the Image J software (“Manual Tracking” plug-in) (shown in Figure 4B). Instant velocity was analyzed for each cell type in Figure 4B and plotted as a function of time (0–10 hours). (**B**) B16-F10 cells transfected with pLacIOP (mock), pLacIOP caveolin 1/Y14F mutant (Y14F), or a clone (clone 3) obtained from cells transfected with pLacIOP caveolin 1 (WT) (described in Figure 4) were treated with 1mM IPTG for 24 hours. Then, cell migration was assessed in a Boyden chamber assay by seeding cells (5×104) on fibronectin coated (2 µg/ml) transwell plates and allowing migration for 2 hours. Cells that migrated to the lower side were detected by crystal violet staining. Bottom panels show total protein extracts, separated by SDS PAGE (35 µg total protein per lane) and analyzed by Western Blotting. Data are representative of two independent experiments.
(TIF)

Figure S4 **Effect of the caveolin-1/Y14F mutant on focal adhesion disassembly.** B16-F10 cells transfected with either pLacIOP (mock) or pLacIOP-caveolin-1/Y14F mutant (Y14F) were seeded onto fibronectin-coated coverslips (2 µg/ml), grown in the presence of 1 mM IPTG for 24 hours and treated with 10 µM nocodazole in serum-free medium for 4 hours. Then, nocodazole was removed by wash-out with serum-free medium and cells were incubated by 0 and 60 min at 37°C. Subsequently, cells were fixed and stained with anti-vinculin antibody (red) and DAPI (blue) to label FAs and nuclei, respectively. Images shown are representative of results from three independent experiments. FAs were quantified by using the Image J software (see Figure 6E).
(TIF)

Figure S5 **Effect of caveolin-1 on cell adhesion.** MDA-MB-231 cells treated with shRNA-control (ctrl) or shRNA-caveolin-1 (sh-cav1) and B16-F10 cells transfected with pLacIOP (mock) or pLacIOP-caveolin-1 (cav1) were held in suspension and allowed to attach to fibronectin-coated plates (2 µg/ml). Cell adhesion was monitored at different time points by crystal violet staining. Data are representative of two independent experiments in triplicate (mean ± SD).
(TIF)
